# Relationship Between Carotid Artery Anatomy and Geometry and White Matter Hyperintensities and Accompanying Comorbid Factors

**DOI:** 10.3390/tomography12010012

**Published:** 2026-01-22

**Authors:** Semih Sağlık, Ayfer Ertekin

**Affiliations:** 1 Department of Radiology, Siirt Training and Research Hospital, Siirt 56100, Turkey; 2 Department of Neurology, Siirt Training and Research Hospital, Siirt 56100, Turkey

**Keywords:** internal carotid artery, carotid bifurcation, white matter hyperintensity (WMH), tortuosity index (TI)

## Abstract

Background/Objectives: This study aimed to investigate the relationship between carotid artery anatomy and geometry and white matter hyperintensities (WMH) and to determine whether it is a risk factor for the disease. Methods: The geometry and anatomy of both carotid arteries were evaluated with the three-dimensional vessel model obtained from the computed tomography angiography (CTA) data, and the segmentation software calculated the geometrical features of the arteries. In this model, vascular diameter, vascular cross-sectional area, carotid bifurcation and internal carotid artery (ICA) angles, as well as ICA tortuosity index (TI) measurements of the common carotid artery (CCA) and ICA were determined. Results: Compared with the non-WMH group, increased carotid bifurcation and ICA angle and higher ICA TI values were found in the WMH group (*p* < 0.001). In multivariate regression analysis, increased carotid bifurcation angle, higher ICA TI values, age, hypertension, and stroke history were identified as independent risk factors for the development of WMH (*p* < 0.05). In addition, age, carotid bifurcation angles and ICA angles were found to be associated with the severity of WMH (*p* < 0.05). Conclusions: Considering the vascular pathologies involved in the pathogenesis of WMH, identifying these risk factors may help determine individuals who are at an increased risk.

## Simple Summary

White matter hyperintensities (WMH) are common brain imaging findings and are associated with aging and vascular risk factors. This study examined whether anatomical and geometrical features of the carotid arteries are related to the presence and severity of WMH. Using three-dimensional models derived from computed tomography angiography, we found that increased carotid bifurcation and internal carotid artery angles, as well as higher internal carotid artery tortuosity, were more frequent in individuals with WMH. These vascular features, together with age, hypertension, and prior stroke, were identified as independent risk factors for WMH. Our findings suggest that carotid artery geometry may play a role in the development and progression of WMH and could help identify individuals at higher risk.

## 1. Introduction

White Matter Hyperintensity (WMH) is a small vessel disease that is identified in the periventricular or subcortical regions during brain magnetic resonance imaging (MRI), particularly in individuals with hypertension and the elderly population [1]. WMH may increase the likelihood of stroke and negatively affect the clinical outcomes of patients after stroke. During the post-stroke recovery process, white matter hyperintensities may complicate the recovery of cognitive functions and may disrupt the rehabilitation process [2]. WMH has been reported to be associated with an increased risk of stroke, dementia, and death. Early diagnosis and intervention can help prevent or slow the progression of this condition [3]. The pathogenesis of WMH is not yet well understood. Lipohyalinosis, arteriolosclerosis, fibrinoid necrosis, and chronic hypoperfusion are among the mechanisms that play an important role in the development of WMH. All these processes cause chronic hypoperfusion and ischemia, leading to perivascular edema and secondary brain damage, accelerating the development of hyperintensities in the white matter [4].

Arterial geometry has a three-dimensional structure, and this geometric structure plays a critical role in arterial blood flow and functionality. Carotid arterial geometry can influence the speed, direction, and distribution of blood flow, leading to alterations in the internal structure of the arteries [5]. Although both carotid arteries are exposed to the same risk factors, they can exhibit different vascular pathological features. This situation can be influenced by factors such as the genetic structure of individuals, vascular anatomy, and hemodynamic conditions. This situation shows that the anatomical and geometric parameters of the carotid artery may predispose to certain vascular pathologies [6,7]. In the literature, most research on carotid geometry has focused primarily on the pathophysiology of vascular diseases, such as atherosclerosis and stroke. However, to our knowledge, there are very few studies in the current literature examining the relationship between carotid artery geometry and WMH. Anatomical parameters, such as carotid artery diameter, cross-sectional area, wall thickness, and tortuosity, may influence blood flow to the brain tissue by affecting blood flow dynamics. Therefore, we hypothesized that anatomical and geometrical features of the carotid artery may potentially affect white matter hyperintensities.

Our study aimed to investigate the relationship between carotid artery anatomy and geometry and WMH and to determine whether it is a risk factor for the disease.

## 2. Materials and Methods

### 2.1. Study Population

All procedures followed the Declaration of Helsinki of 1975, as revised in 2008. Ethical approval for this study was obtained from the Siirt University Non-Invasive Ethics Committee (approval number: 131081, dated 28 January 2025), and informed consent has been obtained from all participants.

This study retrospectively analyzed the registry data of patients admitted to our hospital with various cerebrovascular symptoms and complaints between 2020 and 2025 and evaluated by relevant clinical specialists. A total of 1820 patients who underwent both cervical computed tomography angiography (CTA) and brain MRI during the same admission were assessed, and 468 patients over the age of 18 who met the inclusion criteria were included in the study. In this study, patients were excluded from the analysis in cases of artifacts or poor image quality in MRI or CT images, presence of large infarcts or hemorrhages in the brain parenchyma, presence of degenerative neurological diseases, presence of stents in the carotid arteries or patients with a history of surgery, tumors, head trauma or known connective tissue diseases.

### 2.2. CTA Imaging Protocol

CTA imaging was performed with a 64-slice GE Revolution Evo device. The parameters used during acquisition were as follows: 300 mA, 100 kVp, angular distance (pitch) of 0.625, slice thickness of 1 mm, and slice acquisition interval of 0.5 mm. Contrast material was applied at a rate of 3.5–4.0 mL/s with the help of automatic injection, and images from the ascending aorta to the level of the internal carotid artery (ICA) bifurcation were obtained.

### 2.3. Three-Dimensional Reconstruction of Carotid Artery

Images were transferred to a semi-automatic workstation (AW 4.7 version, GE Healthcare, Chicago, IL, USA) for the analysis of carotid arteries. During the image processing phase, methods such as thresholding, region enlargement, and the separation mask function were employed. These techniques help clean the unwanted background in the image, define specific tissues more clearly, and separate different structures. Three-dimensional models were obtained in the vascular reconstruction starting from the aortic arch and extending along both common carotid arteries (CCA) and ICA. A center point was determined for each of the geometric parameters to capture the flow dynamics within the carotid artery loops and branches precisely.

### 2.4. Measurement of Carotid Artery Geometry

**Figure 1:**
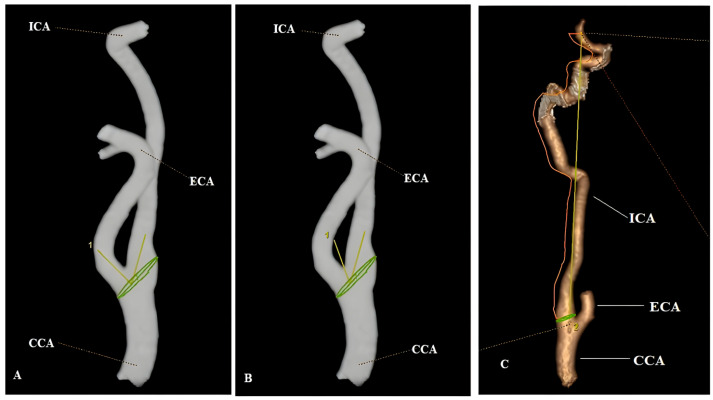
Carotid bifurcation angle (**A**) and ICA angle (**B**) measurements on the 3D arterial model created on the semi-automatic workstation. Measurement of vascular tortuosity (**C**): calculated as the ratio of the true lumen path from the ICA origin to the bifurcation to the distance between these endpoints (orange line; true lumen path, yellow line; distance between the two endpoints). Note: CCA; common carotid artery, ICA; internal carotid artery, ECA; external carotid artery.

After reconstructing the 3D image with maximum intensity projection and volume rendering techniques, the geometry and anatomy of both carotid arteries were evaluated with the three-dimensional vessel model obtained from CTA data, and the geometric features of the arteries were calculated by segmentation software. The central lumen lines determined along the carotid artery lumens allowed the creation of vascular radii. The bifurcation plane was determined using the central line paths with the vascular radii. The diameters and cross-sectional areas of each vascular artery were measured 2 cm from the carotid bulbus utilizing the central lines. The carotid bifurcation angle was determined as the angle between the central vector projections passing through the ICA and ECA lumens from the carotid bulbus plane. The ICA angle was formed by the CCA and ICA vectors located in the carotid bifurcation plane. The ratio of the actual lumen path from both ICA origins to the bifurcation to the distance between these endpoints was defined as the tortuosity index (TI) [8]. For each patient, the diameter, area, angle, and TI values of the bilateral carotid arteries were calculated separately and averaged (Figure 1).

### 2.5. Measurement of Arterial Stenosis

The degree of ICA stenosis was calculated using a three-dimensional vessel model derived from CTA data, in accordance with the North American Symptomatic Carotid Endarterectomy Trial (NASCET) criteria [4]. Since carotid stenosis causes secondary changes in vascular anatomy, individuals with stenosis of 30% or more were excluded from the study to minimize these secondary effects [9]. As a result, patients were categorized according to the presence of plaque: those without plaque in both carotid arteries and those with plaque in at least one carotid artery. Additionally, patients were categorized based on the presence of calcification in the aortic arch.

### 2.6. MRI Examination and Analysis

**Figure 2:**
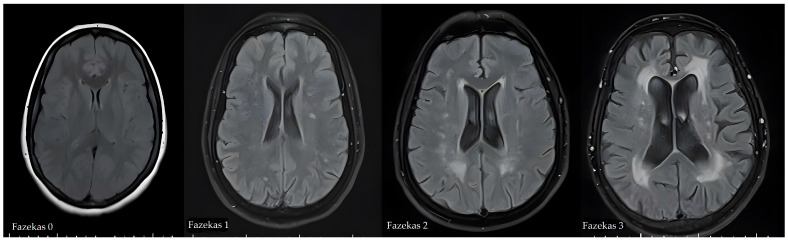
Axial FLAIR images showing the classification of white matter hyperintensities on the four stage Fazekas scale: the healthy brain (Fazekas 0) contrasted with punctiform (Fazekas 1), early confluent (Fazekas 2) and diffuse confluent (Fazekas 3) white matter hyperintensities.

Magnetic resonance imaging examinations were performed using a 1.5T MRI system (MAGNETOM Altea 1.5T, Siemens, Amberg, Germany). The parameters were as follows: (i) TR/TE = 6130/87 ms, slice thickness = 5 mm, and FOV = 190 × 330 mm for T2WI; (ii) TR/TE = 3850/82 ms, slice thickness = 5 mm, and FOV = 190 × 320 mm for FLAIR imaging; and (iii) TR/TE = 5900/85 ms, slice thickness = 5 mm, FOV = 220 × 220 mm; (iv) three directions of diffusion gradient and two b-values (0 and 1000 mm^2^/s) for DWI. T2 FLAIR MRI images of all individuals were evaluated by a radiologist with 15 years of experience. WMHs were graded according to the Fazekas scale (grades 0–3) based on MRI findings [10]. White matter hyperintensities were classified on the four-stage Fazekas scale as: healthy brain (Fazekas 0), punctate (Fazekas 1), early confluent (Fazekas 2), and diffuse confluent (Fazekas 3) (Figure 2). In individuals with Fazekas grades 1–2, small and predominantly negative effect sizes were observed across all cognitive domains compared with healthy controls, indicating only minimal deviation from normal cognitive functioning. In contrast, the pattern of cognitive impairment becomes more pronounced at the Fazekas 3 level [11]. Therefore, in our study, cases were grouped by the severity of WMH, with Fazekas grades 1 and 2 combined into a single group.

### 2.7. Other Study Variables

Hypertension (HT) was defined as systolic/diastolic blood pressure values of 140/90 mmHg or higher or the use of antihypertensive medication. Diabetes mellitus (DM) was defined as a fasting blood sugar level ≥ 7.0 mmol/L or the use of hypoglycemic agents. Hyperlipidemia was described as a low-density lipoprotein (LDL) cholesterol level of 4.1 mmol/L or higher, a total cholesterol level of 6.2 mmol/L or higher, or the use of antilipemic drugs. Stroke was diagnosed by a known history of stroke or the presence of ischemic lesions detected on brain imaging. Coronary artery disease (CVD) was determined by angiography or a history of previous myocardial infarction. Patients diagnosed with epilepsy, Alzheimer’s, Parkinson’s, migraine, thyroid or psychiatric diseases or using medications for these diseases were identified.

### 2.8. Statistical Analysis

SPSS 20.0 software (Statistical Package for Social Sciences, Chicago, IL, USA) was used in the data analysis process. Qualitative data were presented in the form of number (*n*) and percentage (*%*); quantitative data were expressed as mean ± standard deviation (SD).

During data analysis, Student’s *t* test was applied for variables with normal distribution, and Mann–Whitney U test was applied for variables without normal distribution in comparisons between groups. The chi-square test or Fisher’s exact test was used for analyzing relationships between categorical data, depending on the sample size. Based on the relationships identified in the multivariate analysis, post hoc analyses were conducted to examine the associations between carotid anatomy and geometry and clinical, radiological, and demographic factors. Univariate and Multivariate Binary Logistic Regression analyses were performed to determine risk factors for WMH. The significance level in statistical analyses was defined as *p* < 0.05.

## 3. Results

According to MRI results, patients are divided into two groups based on the presence of WMH, and the carotid geometric imaging results are summarized in Table 1. Patients with WMH (67.7 ± 13.5) were older than those without WMH (53.3 ± 17.7), and a significant age difference was observed between the two groups (*p* < 0.001). Patients with WMH had higher rates of HT (74.4% vs. 25.6%), DM (68.8% vs. 31.2%), hyperlipidemia (73.7% vs. 26.3%), CVD (72.9% vs. 27.1%), and history of stroke (75.9% vs. 24.1%) (*p* < 0.05). However, aortic arch calcification (79.6% vs. 20.4%) and carotid artery stenosis (82.6% vs. 17.4%) detected on CT scans in the group with WMH were significantly higher than in the other group (*p* < 0.001). When the carotid geometric parameters of both groups were compared, higher bifurcation angle (55.58 ± 19.43 vs. 45.18 ± 0.96), ICA angle (27.08 ± 15.3 vs. 21.55 ± 9.5), and ICA TI values (1.87 ± 0.51 vs. 1.55 ± 0.35) were detected in the group with WMH (*p* < 0.001).

**Figure 3:**
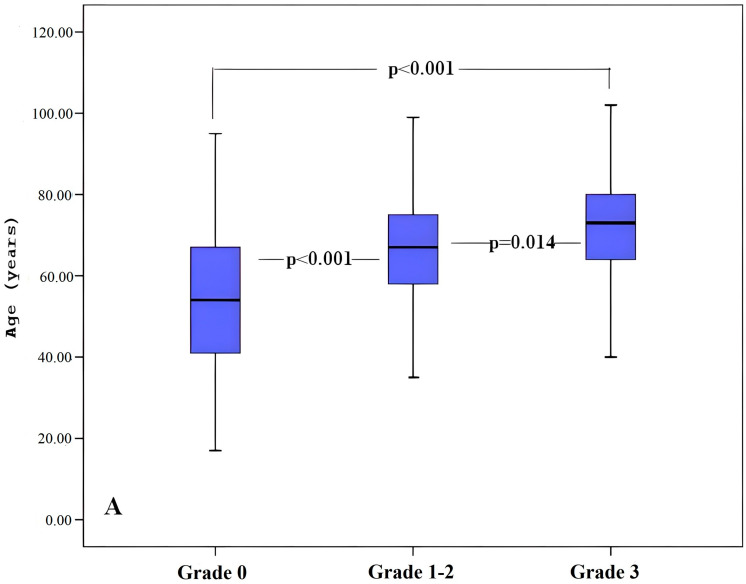

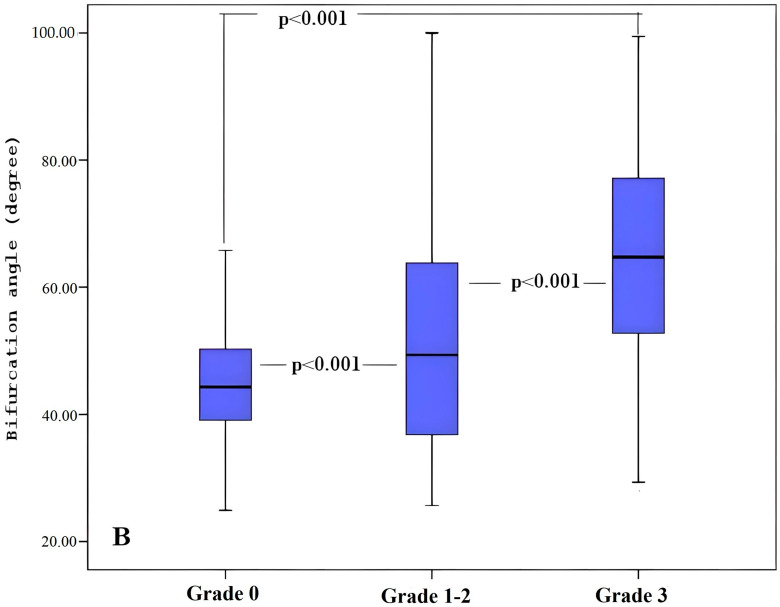

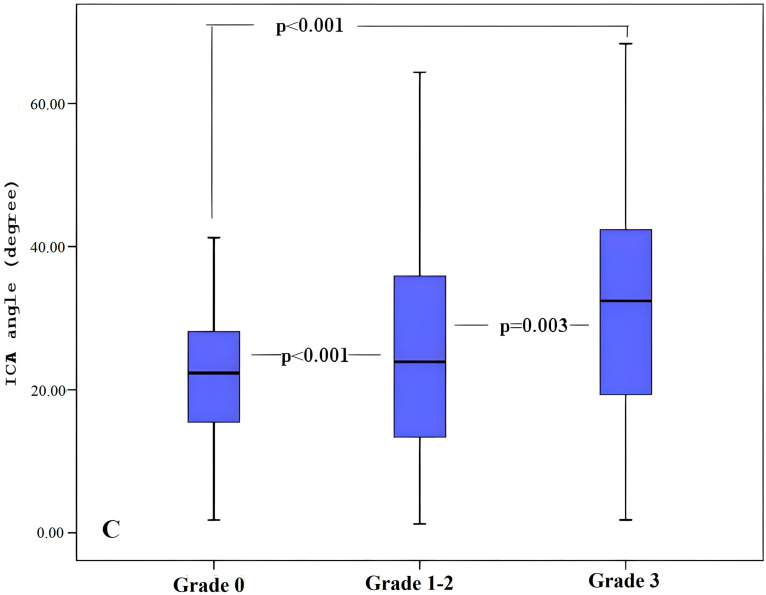

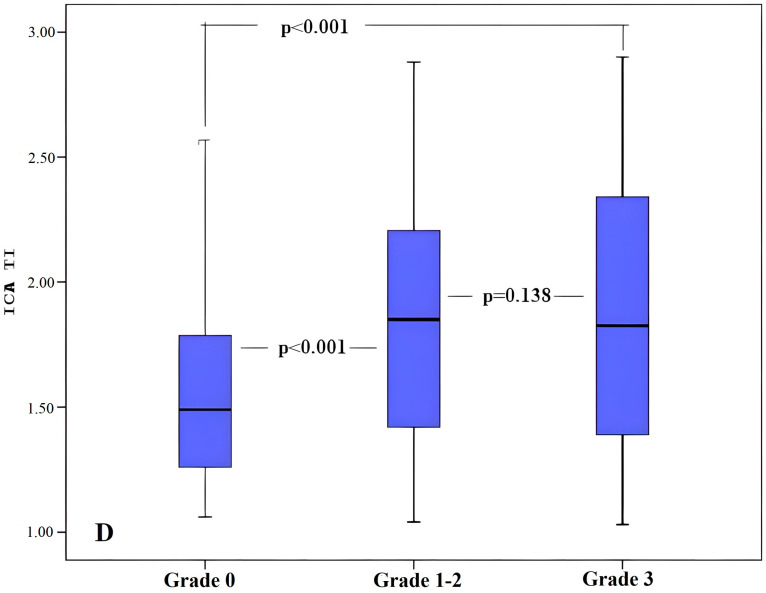
Relationship between age (**A**), carotid bifurcation angle (**B**), ICA angle (**C**) and ICA TI (**D**) values and the severity of white matter hyperintensities. Horizontal lines within each box represent mean values, and lower and upper lines of each box represent minimum and maximum values, respectively. Vertical lines and whiskers indicate 95% CIs. Note: ICA; internal carotid artery, TI; tortuosity index, Grade; Fazekas scale.

An ANOVA test was applied to determine the parameters associated with WMH severity, followed by a Tukey post hoc test to identify the direction of the differences. According to the test results, a linear relationship was found between WMH severity and age, as well as between carotid bifurcation angle and ICA angle (*p* < 0.05) (Table 2 and Figure 3).

Regression analysis was used to determine risk factors in patients with WMH diagnosis. In univariate regression analysis, age (OR, 1.06 per year increment; 95% CI, 1.04–1.07), hypertension (OR, 3.77; 95% CI, 2.54–5.59), diabetes mellitus (OR, 1.58; 95% CI, 1.05–2.37), hyperlipidemia (OR, 2.05; 95% CI, 1.29–3.26), CVD (OR, 2.17; 95% CI, 1.44–3.26), stroke history (OR, 3.02; 95% CI, 2.02–4.52), aortic arch calcification (OR, 4.07; 95% CI, 2.67–6.20), carotid artery stenosis (OR, 4.41); ICA angle (OR, 1.03 per degree increment; 95% CI, 1.01–1.04), bifurcation angle (OR, 1.04 per degree increment; 95% CI, 1.02–1.05) and ICA TI (OR, 0.46 per unit increment; 95% CI, 0.28–0.73) were determined as risk factors. In multivariate regression analysis, age (OR, 1.02 per year increment; 95% CI, 1.01–1.04), hypertension (OR, 1.94; 95% CI, 1.15–3.26), history of stroke (OR, 2.32; 95% CI, 1.44–3.76), bifurcation angle (OR, 1.03 per degree increment; 95% CI, 1.01–1.05) and ICA TI (OR, 1.25 per unit increment; 95% CI, 1.14–1.44) were determined as independent risk factors (Table 3).

## 4. Discussion

We have demonstrated that carotid anatomy and geometry play an independent role in the development of white matter hyperintensity lesions in patients with MRI-confirmed white matter hyperintensity lesions. Higher carotid bifurcation angle and increased ICA TI values may lead to hemodynamic changes in the carotid artery. These hemodynamic changes may contribute to the development of WMH and contribute to the formation of these lesions. In addition to traditional risk factors, identifying these individual risk factors may help identify patients at high risk of developing WMH much earlier.

Abrupt changes in carotid bifurcation geometry and anatomy may cause blood flow disturbances and turbulence, leading to various vascular complications [12]. A portion of the pulse wave reaching the bifurcation is reflected; as the degree of reflection increases, local hemodynamic stress and flow energy loss also increase [12]. However, the hemodynamic mechanisms contributing to the structural brain lesions and cognitive impairments associated with these vascular changes remain unclear [13]. Mitchell et al. hypothesized that changes in the aorta and carotid artery structure reduce flow wave reflection and, as a result, facilitate the transmission of excessive pulsatile energy to the cerebral microcirculation, leading to microvascular damage and dysfunction [13]. Our study findings indicate a significant relationship between WMH and the carotid bifurcation angle and the ICA angle, with the severity of WMH increasing as the bifurcation angle increases. Possible reasons for this situation may be the loss of normal flow level due to disruption of the normal anatomical and geometric structure of the carotid arteries, resulting in increased tension on the vessel walls [6]. This may cause perivascular edema and secondary brain damage [14]. In addition, to our knowledge, our study is the first to examine the relationship between WMH and the carotid bifurcation angle. However, we did not find a significant relationship between the diameter and area measurements of the carotid arteries and WMH in our study. A possible reason for this may be that our study excluded patients with moderate and high-grade stenosis in the carotid arteries because carotid stenosis can lead to secondary changes in vascular anatomy [12].

Our study results show that ICA TI is significantly associated with WMH lesions. Studies have shown that arterial tortuosity is associated with cerebral minor vessel diseases, but there are only a few studies examining its relationship with white matter hyperintensities [15,16,17]. In our research, since there is no definitive diagnostic criterion for ICA vessel tortuosity, we preferred the TI value as a more straightforward and more practical method. Arterial tortuosity can lead to a loss of vascular elasticity and increased pulsatility, as reflected in the brain parenchyma [10]. Therefore, changes in the tortuosity of the carotid artery may play a crucial role in the formation of abnormal hemodynamic forces that contribute to the development of WMH lesions. Our findings show that ICA TI values are an independent risk factor for WMH, but they are not associated with its severity. Chen et al. stated in their study that arterial tortuosity is only associated with grade 3 white matter hyperintensities [10]. This inconsistency may be due to differences in the research group and the methodology used. In the past,

WMH was often considered a natural consequence of advancing age and was not taken seriously. However, in recent years, large-scale studies have shown that this is not a universally held belief and that, although it increases with age, its prevalence remains relatively constant across all age groups. More importantly, studies have emphasized that this condition should be taken seriously due to its relationship with significant clinical and risk factors [18]. WMH is strongly associated with cognitive impairment, dementia, stroke, depression, balance disorders, and poor physical performance, and it increases the risks of these diseases [18,19,20]. The main determinants of WMH severity are age and HT, as well as many other risk factors such as DM, smoking, CVD, and hyperlipidemia [1,18,19].

The large sample size in our study allowed us to comprehensively evaluate the risk factors associated with WMH. Our current findings are consistent with previous studies, which have shown that high blood pressure is associated with WMH [21,22]. We found that hypertension is an independent risk factor for WMH. Hypertension, which is identified as the primary risk factor for WMH, along with age, is recognized as the most significant risk factor for minor vessel diseases [21]. Cerebral small vessel disease caused by hypertension and hemodynamic damage in the blood–brain barrier may lead to the development of WMH [23].

Current studies indicate that aging is the most significant risk factor for WMH [24]. Numerous studies demonstrate a strong correlation between age and WMH, indicating that aging makes an independent contribution to the prevalence and severity of WMH [20,25]. Our study findings, in line with the existing literature, demonstrate that age is a significant independent risk factor for WMH and is also associated with its severity.

The relationship between DM and WMH remains unclear. Some cross-sectional studies have suggested that there is no clear relationship between diabetes mellitus and white matter hyperintensities [26,27]. However, some studies contradict this view, arguing that a relationship exists between DM and WMH, particularly in elderly individuals [28,29]. Our study findings also revealed a statistically significant relationship between DM and WMH. Reactive oxygen species generated by diabetes induce oxidative stress, which negatively impacts the endothelium and impairs its function [30]. The microvascular abnormalities associated with this condition may trigger or exacerbate the formation of WMH in the brain [31].

Rost et al. reported that WMH load was higher in patients with acute cerebral infarction and that WMH was an independent predictor of cerebrovascular disease [32]. Ghaznawi et al. argued that WMH volume and shape may be associated with mortality and poor prognosis in stroke patients [33]. In addition, in addition to the negative effects of WMH on cognitive functions, it has also been associated with the volume, recurrence, and severity of infarction [1,34]. It has been suggested that this may be related to impaired vascular density and cerebral blood flow in WMH regions [1,35]. The results of this study were also consistent with the existing literature, showing that WMH is associated with stroke and may even be an independent risk factor.

The relationship between hyperlipidemia and WMH is inconsistent in the literature [21,24]. Although there are some studies suggesting that hyperlipidemia is a risk factor for WMH, there are also studies that do not support this view [20,36]. Our study findings showed that hyperlipidemia may be a risk factor for WMH.

The anatomical structure and geometric characteristics of the carotid artery may influence local hemodynamic flow patterns within the vascular lumen, alter wall shear stress, and thereby contribute to the development of carotid artery atherosclerosis. Atherosclerosis in large arteries such as the aorta and carotid arteries has also been reported to be associated with white matter hyperintensities (WMH) [25,37]. The reason for this association is explained by the hardening and narrowing of the vessels caused by atherosclerosis, which negatively affects cerebral perfusion by reducing blood flow to the brain tissue, thereby impairing the flow ability of the cerebral vessels [25]. Increased pulse pressure caused by atherosclerosis in large arteries may be reflected in the pressure-sensitive cerebral small vessels, leading to the development of WMH [25]. Some studies have reported that the prevalence of WMH increases in individuals with coronary artery disease and that cardiovascular plaques may be a risk factor for WMH [38,39]. Our current findings also suggest that cardiovascular diseases may be a risk factor for WMH.

## 5. Conclusions

In conclusion, our study findings indicate that, in addition to HT and age, which are the primary risk factors for WMH, ICA TI and carotid bifurcation angle are also independent risk factors for this disease. Given the vascular pathologies involved in the pathogenesis of WMH, identifying these risk factors may help identify individuals with an increased risk.

## 6. Limitations

This study has several limitations. First, the use of a retrospective and cross-sectional design may create selection bias. Second, since radiological imaging was performed according to clinical requirements, individuals included in the study may have higher vascular risk. However, excluding patients with carotid artery stenosis greater than 30% contributed to minimizing this effect. Third, since the smoking status and body mass index data of the individuals included in the study were not recorded in the system, the relationship between these risk factors and WMH was not evaluated. Finally, since our study group represents a specific region and ethnic group, our results do not necessarily reflect the characteristics of the general population. Therefore, our study findings should be supported by future multicenter studies with a large population.

## Figures and Tables

**Table 1:** Demographic data and imaging factors among individuals with and without White Matter Hyperintensity.

	White Matter Hyperintensity			
Parameters	Negative (*n* = 179)	Positive (*n* = 289)	Total (*n* = 468)	*p* Values
Age (years) ± SD	53.3 ± 17.7	67.7 ± 13.5	62.2 ± 16.7	<0.001 ^a^
Gender, *n* (%) Female Male	84 (37.2%) 95 (39.3%)	142 (62.8%) 147 (60.7%)	226 (48.3%) 242 (51.7%)	0.704 ^b^
DM, *n* (%) No Yes	130 (41.8%) 49 (31.2%)	181 (58.2%) 108 (68.8%)	311 (66.5%) 157 (33.5%)	0.027 ^b^
HT, *n* (%) No Yes	108 (56.5%) 71 (25.6%)	83 (43.5%) 206 (74.4%)	191 (40.8%) 277 (59.2%)	<0.001 ^b^
Hyperlipidemia, *n* (%) No Yes	148 (42.3%) 31 (26.3%)	202 (57.7%) 87 (73.7%)	350 (74.8%) 118 (25.2%)	0.002 ^b^
Stroke History, *n* (%) No Yes	130 (49.1%) 49 (24.1%)	135 (50.9%) 154 (75.9%)	265 (56.6%) 203 (43.4%)	<0.001 ^b^
CVD, *n* (%) No Yes	133 (44.6%) 46 (27.1%)	165 (55.4%) 124 (72.9%)	298 (63.7%) 170 (36.3%)	<0.001 ^b^
Alzheimer’s disease, *n* (%) No Yes	160 (39.6%) 19 (10.6%)	244 (60.4%) 45 (15.6%)	404 (86.3%) 64 (13.7%)	0.129 ^b^
Parkinson’s disease, *n* (%) No Yes	177 (38.9%) 2 (1.1%)	278 (61.1%) 11 (3.8%)	455 (97.2%) 13 (2.8%)	0.085 ^c^
Epilepsy disease, *n* (%) No Yes	167 (38.0%) 12 (41.4%)	272 (62.0%) 17 (58,6%)	439 (93.8%) 29 (6.2%)	0.720 ^b^
Psychiatric disease, *n* (%) No Yes	147 (36.8%) 32 (46.4%)	252 (63.2%) 37 (53.6%)	399 (85.3%) 69 (14.7%)	0.132 ^b^
PAD, *n* (%) No Yes	175 (38.5%) 4 (2.2%)	280 (61.5%) 9 (3.1%)	455 (97.2%) 13 (2.8%)	0.574 ^c^
Thyroid disease, *n* (%) No Yes	169 (38.4%) 10 (10.7%)	271 (61.6%) 18 (17.3%)	440 (94.0%) 28 (6.0%)	0.776 ^b^
Migraine disease, *n* (%) No Yes	165 (36.7%) 14 (7.8%)	284 (63.3%) 5 (1.7%)	449 (95.9%) 19 (4.1%)	0.456 ^c^
Aortic arch plaque, *n* (%) No Yes	139 (51.1%) 40 (20.4%)	133 (48.9%) 156 (79.6%)	272 (58.1%) 196 (41.9%)	<0.001 ^b^
Carotid artery stenosis, *n* (%) No Yes	153 (48.3%) 26 (17.4%)	164 (51.7%) 123 (82.6%)	317 (68.0%) 149 (32.0%)	<0.001 ^b^
CCA diameter (mm) ± SD	6.77 ± 1.15	6.92 ± 1.13	6.86 ± 1.13	0.184 ^d^
Bifurcation diameter (mm) ± SD	11.19 ± 1.87	11.27 ± 1.88	11.24 ± 1.88	0.654 ^d^
ICA diameter (mm) ± SD	5.28 ± 0.73	5.42 ± 0.72	5.37 ± 0.73	0.035 ^d^
CCA area (mm^2^) ± SD	41.56 ± 6.36	42.69 ± 6.4	42.26 ± 6.41	0.063 ^d^
Bifurcation area (mm^2^) ± SD	79.91 ± 14.16	79.97 ± 15.1	79.94 ± 14.70	0.967 ^d^
ICA area (mm^2^) ± SD	22.91 ± 4.43	22.44 ± 4.53	22.62 ± 4.5	0.272 ^d^
Bifurcation angle (degree) ± SD	45.18 ± 8.96	55.58 ± 19.43	51.6 ± 17	<0.001 ^d^
ICA angle (degree) ± SD	21.55 ± 9.5	27.08 ± 15.3	24.96 ± 13.64	<0.001 ^d^
ICA TI ± SD	1.55 ± 0.35	1.87 ± 0.51	1.78 ± 0.49	<0.001 ^d^

Notes: ^a^ Student’s *t*-test with mean ± standard deviation (SD). ^b^ Chi-Square with *n* (%).^c^ Fisher’s Exact test with *n* (%). ^d^ Mann–Whitney U-test with median ± interquartile range (IQR). Statistically significant results (*p* < 0.05). Abbreviations: SD; Standard deviation, PAD, peripheral arterial disease, DM, Diabetes Mellitus, HT; Hypertension; CVD; Cardiovascular Disease, TI; Tortuosity index, CCA; Common Carotid Artery, ICA; internal carotid artery.

**Table 2:** The severity of White Matter Hyperintensities and carotid geometry variables.

Parameters	Group 1	Group 2	Group 3	*p* Values
Age (years) ± SD Group 1 vs. 2 Group 1 vs. 3 Group 2 vs. 3	53.36 ± 17.7	66.12 ± 13.5	71.69 ± 12.95	<0.001 ^a^ <0.001 ^b^ <0.001 ^b^ 0.014 ^b^
Bifurcation angle (degree) ± SD Group 1 vs. 2 Group 1 vs. 3 Group 2 vs. 3	45.18 ± 8.96	52.04 ± 18.68	64.51 ± 18.5	<0.001 ^a^ <0.001 ^b^ <0.001 ^b^ <0.001 ^b^
ICA angle (degree) ± SD Group 1 vs. 2 Group 1 vs. 3 Group 2 vs. 3	21.55 ± 9.5	25.47 ± 14.91	31.12 ± 15.63	<0.001 ^a^ 0.011 ^b^ <0.001 ^b^ 0.003 ^b^
ICA TI ± SD Group 1 vs. 2 Group 1 vs. 3 Group 2 vs. 3	1.55 ± 0.35	1.85 ± 0.51	1.96 ± 0.52	<0.001 ^a^ <0.001 ^b^ <0.001 ^b^ 0.138 ^b^

Note: ^a^ *p* < 0.05 was considered statistically significant (one-way ANOVA). ^b^ *p* < 0.05 was considered statistically significant (one-way ANOVA with a post hoc Tukey test). Abbreviations: SD; Standard deviation, TI; Tortuosity index, ICA; Internal Carotid Artery, Group 1; Fazekas 0; Group 2; Fazekas 1–2, Group 2; Fazekas 3.

**Table 3:** Univariate and Multivariate Binary Logistic Regression Analysis Results to Determine Risk Factors Effective in White Matter Hyperintensity.

	Univariate		Multivariate	
	*p* Values	OR (CI 95%)	*p* Values	OR (CI 95%)
Age (years) per year increment	<0.001	1.06 (1.04–1.07)	0.005	1.02(1.01–1.04)
DM yes against no	0.027	1.58 (1.05–2.37)	0.252	ns
HT yes against no	<0.001	3.77 (2.54–5.59)	0.012	1.94 (1.15–3.26)
Hyperlipidemia yes against no	0.002	2.05 (1.29–3.26)	0.088	ns
CVD yes against no	<0.001	2.17 (1.44–3.26)	0.125	ns
Stroke History yes against no	<0.001	3.02 (2.02–4.52)	0.001	2.32 (1.44–3.76)
Aortic arch plaque yes against no	<0.001	4.07 (2.67–6.20)	0.067	ns
Carotid artery stenosis yes against no	<0.001	4.41 (2.73–7.11)	0.354	ns
Bifurcation angle per degree increment	<0.001	1.04 (1.02–1.05)	0.001	1.03 (1.01–1.05)
ICA angle per degree increment	<0.001	1.03 (1.01–1.04)	0.096	ns
ICA TI per unit increment	<0.001	1.46 (1.28–1.73)	0.001	1.25 (1.14–1.44)

Note: Statistically significant results (*p* < 0.05). Abbreviations: ns, not significant; OR, Odds ratio; CI, Confidence interval; DM, Diabetes Mellitus, HT; Hypertension; CVD; Cardiovascular Disease, TI; Tortuosity index, ICA; Internal Carotid Artery.

## Data Availability

The original contributions presented in this study are included in the article. Further inquiries can be directed to the corresponding author.

## Abbreviations

The following abbreviations are used in this manuscript:

WMH	White Matter Hyperintensities
MRI	Magnetic Resonance Imaging
CTA	Computed Tomography Angiography
ICA	Internal Carotid Artery
CCA	Common Carotid Arteries
TI	Tortuosity Index
